# Mobile and Web-Based Partnered Intervention to Improve Remote Access to Pain and Posttraumatic Stress Disorder Symptom Management: Recruitment and Attrition in a Randomized Controlled Trial

**DOI:** 10.2196/49678

**Published:** 2023-10-03

**Authors:** Jolie N Haun, Hari H Venkatachalam, Christopher A Fowler, Amy C Alman, Lisa M Ballistrea, Tali Schneider, Rachel C Benzinger, Christine Melillo, Neil B Alexander, S Angelina Klanchar, William Lapcevic, Dustin D French

**Affiliations:** 1 James A. Haley Veterans' Hospital Research Service Tampa, FL United States; 2 Division of Epidemiology Department of Internal Medicine University of Utah Salt Lake City, UT United States; 3 Department of Psychiatry and Behavioral Neurosciences University of South Florida Tampa, FL United States; 4 College of Public Health University of South Florida Tampa, FL United States; 5 VA Ann Arbor Healthcare System Geriatric Research, Education, and Clinical Center Ann Arbor, MI United States; 6 Division of Geriatric and Palliative Medicine Department of Internal Medicine Ann Arbor, MI United States; 7 Department of Veterans Affairs Center of Innovation for Complex Chronic Healthcare Edward Hines, Jr. VA Hospital Hines, IL United States; 8 Center for Health Services and Outcomes Research Feinberg School of Medicine Northwestern University Chicago, IL United States; 9 Departments of Ophthalmology and Medical Social Sciences Feinberg School of Medicine Northwestern University Chicago, IL United States

**Keywords:** PTSD, pain, veteran, attrition, CIH, randomized controlled trial, chronic pain, remote intervention, dyad, mobile health

## Abstract

**Background:**

Increasing access to nonpharmacological interventions to manage pain and posttraumatic stress disorder (PTSD) is essential for veterans. Complementary and integrative health (CIH) interventions can help individuals manage symptom burden with enhanced accessibility via remotely delivered health care. Mission Reconnect (MR) is a partnered, self-directed intervention that remotely teaches CIH skills.

**Objective:**

The purpose of this paper is to describe the recruitment, onboarding phase, and attrition of a fully remote randomized controlled trial (RCT) assessing the efficacy of a self-directed mobile and web-based intervention for veterans with comorbid chronic pain and PTSD and their partners.

**Methods:**

A total of 364 veteran-partner dyads were recruited to participate in a mixed methods multisite waitlist control RCT. Qualitative attrition interviews were conducted with 10 veterans with chronic pain and PTSD, and their self-elected partners (eg, spouse) who consented but did not begin the program.

**Results:**

At the point of completing onboarding and being randomized to the 2 treatment arms, of the 364 recruited dyads, 97 (26.6%) failed to complete onboarding activities. Reported reasons for failure to complete onboarding include loss of self-elected partner buy-in (n=8, 8%), difficulties with using remote data collection methods and interventions (n=30, 31%), and adverse health experiences unrelated to study activities (n=23, 24%). Enrolled veterans presented at baseline with significant PTSD symptom burden and moderate-to-severe pain severity, and represented a geographically and demographically diverse population. Attrition interviews (n=10) indicated that misunderstanding MR including the intent of the intervention or mistaking the surveys as the actual intervention was a reason for not completing the MR registration process. Another barrier to MR registration was that interviewees described the mailed study information and registration packets as too confusing and excessive. Competing personal circumstances including health concerns that required attention interfered with MR registration. Common reasons for attrition following successful MR registration included partner withdrawal, adverse health issues, and technological challenges relating to the MR and electronic data collection platform (Qualtrics). Participant recommendations for reducing attrition included switching to digital forms to reduce participant burden and increasing human interaction throughout the registration and baseline data collection processes.

**Conclusions:**

Challenges, solutions, and lessons learned for study recruitment and intervention delivery inform best practices of delivering remote self-directed CIH interventions when addressing the unique needs of this medically complex population. Successful recruitment and enrollment of veterans with chronic pain and PTSD, and their partners, to remote CIH programs and research studies requires future examination of demographic and symptom-associated access barriers. Accommodating the unique needs of this medically complex population is essential for improving the effectiveness of CIH programs. Disseminating lessons learned and improving access to remotely delivered research studies and CIH programs is paramount in the post–COVID-19 climate.

**Trial Registration:**

ClinicalTrials.gov NCT03593772; https://clinicaltrials.gov/ct2/show/NCT03593772

## Introduction

### Background

Modern trends in health care, including the COVID-19 pandemic, have resulted in significant changes to health care delivery with an increased focus on remotely accessible, self-directed, and electronically delivered care [1,2]. The Department of Veterans Affairs (VA), a pioneer in remotely delivered health care, leverages existing infrastructure to promote access to remotely delivered treatment options to support veterans and their family members [3,4]. VA is also shifting toward increasing access to complementary and integrative health (CIH) treatments for veterans, particularly those with chronic health conditions [5]. In recent years, a significant amount of CIH health care delivery interventions have been designed to be remotely delivered and self-directed [6], resulting in scientific studies examining outcomes associated with the remote delivery of nonpharmacological CIH treatments.

### Comorbid Chronic Pain and Posttraumatic Stress Disorder

Comorbid chronic pain and posttraumatic stress disorder (PTSD) are highly prevalent among veterans [7,8]. Traditional pharmacological interventions for chronic pain can lead to opioid use disorder, overdose, and even death [9]. The VA’s Opioid Safety Initiative and the US Department of Health and Human Services 2016 National Pain Strategy stress nonpharmacological interventions for chronic pain that emphasize self-management to improve health outcomes. However, empirical evidence and guidelines for the treatment of co-occurring pain and PTSD are lacking [10].

### Partnered Remotely Delivered Nonpharmacological Intervention: Mission Reconnect

Mission Reconnect (MR) is a dyadic CIH intervention for veterans that directs participants in self-management activities of mindfulness and partnered massage [11-13]. Pilot evidence indicated that National Guard veterans had pre-post intervention improvements in PTSD symptoms and pain intensity [11]. In a follow-up multigroup randomized controlled trial (RCT), post-9/11 veterans using MR alone or as a treatment adjunct experienced improvements in PTSD and pain intensity at 8 and 16 weeks from the baseline [12]. MR has been shown to enhance partner relationships to provide care that is acceptable for veterans and partners [11]. Recent VA research also indicates that partner recruitment for remote-delivered interventions is feasible and that their personal relationship with the veteran is a facilitator of recruitment and retention [14]. Furthermore, MR may overcome common treatment barriers including aversion to seeking professional health care services and travel distance for patients [13].

CIH treatments have previously shown effectiveness in reducing symptom burden, improving mobility, and decreasing behavior avoidance for veterans who experience chronic pain and PTSD [15]. The mode of delivery of CIH treatments is commonly believed by veterans to take place in a health care provider setting. MR introduces veterans to a CIH intervention that is delivered in the home setting and performed through self-care activities or dyadic activities between a veteran and their partner [13]. While MR uses self-directed dyadic activities to circumvent barriers associated with seeking in-person treatment and removes barriers associated with transportation, it is unclear whether VHA-wide implementation of MR may be hindered by attrition.

### Remote Interventions and Attrition

Remotely and electronically available interventions and disease monitoring methodologies have significant variability in attrition rates, revealing unique barriers to these interventions compared with traditional in-person treatment options [16,17]. Attrition is also a notable limitation in chronic pain and PTSD intervention studies. Attrition rates for veterans with PTSD range from 12% to 72% across various RCTs and real-world clinical therapies [18-20] and are strongly correlated with comorbidities [21,22]. Similar variability in attrition rates (5%-62%) has been observed in chronic pain intervention studies, even with self-administered and noninvasive interventions [23,24]. High attrition rates can impact the generalizability of findings and threaten the ability to implement interventions in a systems-wide manner [24,25].

### This Study

The purpose of this paper is to describe the recruitment, onboarding, and attrition phases of a fully remote RCT assessing the efficacy of the MR intervention on veterans with comorbid chronic pain and PTSD and their partners. Baseline attrition will inform the acceptability of recruitment for MR, a remotely delivered CIH intervention in a national sample of veteran-partner dyads. Examining baseline attrition can also provide useful insights for determining demographic information, chronic pain and PTSD profiles, and geographic distribution for successfully enrolled participants. This includes considerations for the application of this remotely delivered intervention nationally to a clinically diverse patient population. Recommendations for effective recruitment and onboarding will be provided with lessons learned through qualitative interviews and modifications of the recruitment protocol methodology to assist similar RCTs that recruit from pools of veterans with comorbid chronic pain and PTSD.

## Methods

### Ethics Approval

This study was reviewed for human subject protection and approved (#Pro00035440) by institutional review boards at the universities of South Florida, Michigan, and Washington, respectively. All participants consented prior to study participation, including participants who fell to attrition during the onboarding processes. Participants were compensated with reimbursement for their time. Each participant who provided weekly MR reports on usage and pain ratings for the first 8 weeks received weekly payments of US $5 for a total of US $40. To incentivize study completion, each participant who provided assessment data across each of the 4 time points in a 4-month period received US $20 for a total of US $80. Each telephone interview participant received an additional US $20. The total possible incentive for study participants was up to US $140. All study work was completed in adherence to oversight regulations and the approved published protocol [13]. Study methods and results are reported in accordance with CONSORT-EHEALTH (Consolidated Standards of Reporting Trials of Electronic and Mobile Health Applications and Online Telehealth; Multimedia Appendix 1) [26]. A Data Management and Access Plan was completed and describes the data management and access details for the associated project.

### MR Intervention

The protocol for this RCT has previously been described in detail [13]. MR is a remotely delivered patient-centered self-care management program to help veterans manage their pain and psychological health with their partners. In this RCT, MR was delivered using detailed wellness-based activities including a program overview and detailed massage instruction. Participants were instructed to (1) complete each practice at least once during the first 2 weeks; (2) after week 2, do ≥1 massage exchange weekly with their partner; and (3) practice their preferred methods at ≥3 times weekly [13]. Waitlist control participants were permitted to access MR after the assessment period. Full details of the MR intervention are described elsewhere [11-13].

### Design

This RCT used a mixed design. Treatment arms (MR and waitlist control) were compared between participants. Repeated measurements of patient-reported outcomes (PROs) were assessed among participants.

### Sample Size and Recruitment

This study was conducted at three urban VA hospitals located in the Southeast, Midwest, and Northwest regions of the United States. Sample size estimates were calculated using a conservative 20% (n=46 dyads) attrition rate. Assuming a within-participant correlation of 0.50 for PROs and a type I error rate of 0.05, recruitment of 228 dyads (76 per site) was expected to provide 80% power to detect a small-to-medium effect size (Cohen *d*=0.38) [27]. Protocol modifications for recruitment were made contemporaneously when the attrition rate exceeded expectations. Ultimately, 364 dyads consented to enroll in this study.

Potential participants were identified through secondary administrative data queries of the VA Corporate Data Warehouse (CDW) by identifying veterans with International Classification of Diseases (ICD-10) codes for chronic pain and PTSD who had used VA services at one of the study sites in the 6 months preceding study activities. Veterans with a confirmed diagnosis of chronic pain and PTSD were recruited through targeted mailers. Additional recruitment occurred through presentations at VA facilities, flyers (see Multimedia Appendix 2) disseminated in clinics that may treat comorbidities (ie, chronic pain, PTSD), and media blasts (eg, VA Facebook and website posts), which included study details and eligibility criteria.

Veteran-partner dyads who responded to recruitment efforts were screened to determine whether they met inclusion and exclusion criteria (see Multimedia Appendix 3). Participants who failed to meet study inclusion or exclusion criteria were provided with access to alternative resources. Specifically, the study team provided education on available resources and helped veterans get organized using relevant VA self-management application programs (eg, PTSD Coach and Concussion Coach) [28,29] and CIH programs and resources (MR website, VA Transforming Health and Resiliency through Integration of Values-based Experiences Program) [12,30].

During the study, high attrition rates were identified. In an attempt to iteratively improve recruitment and retainment, attrition interviews were conducted to identify reasons for attrition. The sample size was estimated to require 5-7 interviews to meet saturation based on a single concept (ie, the reason for attrition) with a single cohort (ie, attrition participants) [31]; ultimately, 10 attrition interviews were conducted to ensure saturation.

### Procedure

#### Enrollment

Prospective participants completed a brief in-person or phone screening with a team member to determine eligibility: (1) structured screening interview, (2) informed consent, and (3) Health Insurance Portability and Accountability Act authorization. Partners were also mailed a VA acknowledgment of notice of privacy practices (NOPP) to sign and return. Eligible dyads were then randomized to the MR or waitlist control arms.

#### Randomization

Prior to randomization, participant dyads were stratified by (1) current or recent (past 2 weeks) usage of concurrent treatment strategies for chronic pain and PTSD and (2) recruitment site. To ensure equal group sizes for the MR and waitlist control arms, within strata randomization was conducted using alternating blocks of 6 and 8 dyads. A random number table was generated for use in this study using SAS (version 9.4; SAS Institute Inc). Dyads were initially blinded to treatment assignment to protect against attrition in the control arm. A detailed description of the randomization process for this trial is reviewed in the published protocol [32].

#### MR Onboarding

Participant dyads received access to all study processes via email. Following randomization, participants created a profile on the MR website [33]. Study staff contacted participants regularly to ensure movement through the onboarding process. Participants who stagnated during the onboarding process were contacted up to 3 times to ensure that they received proper guidance for the next steps to complete onboarding. Dyads that successfully completed onboarding were emailed a link to complete baseline measures using Qualtrics, a secure, cloud-based, electronic data collection platform that has demonstrated validity for PRO data collection within the VA system [34]. Following baseline survey completion, assignment to the MR or waitlist control arm was revealed to each dyad.

#### Attrition Classification and Interviews

**Figure 1:**
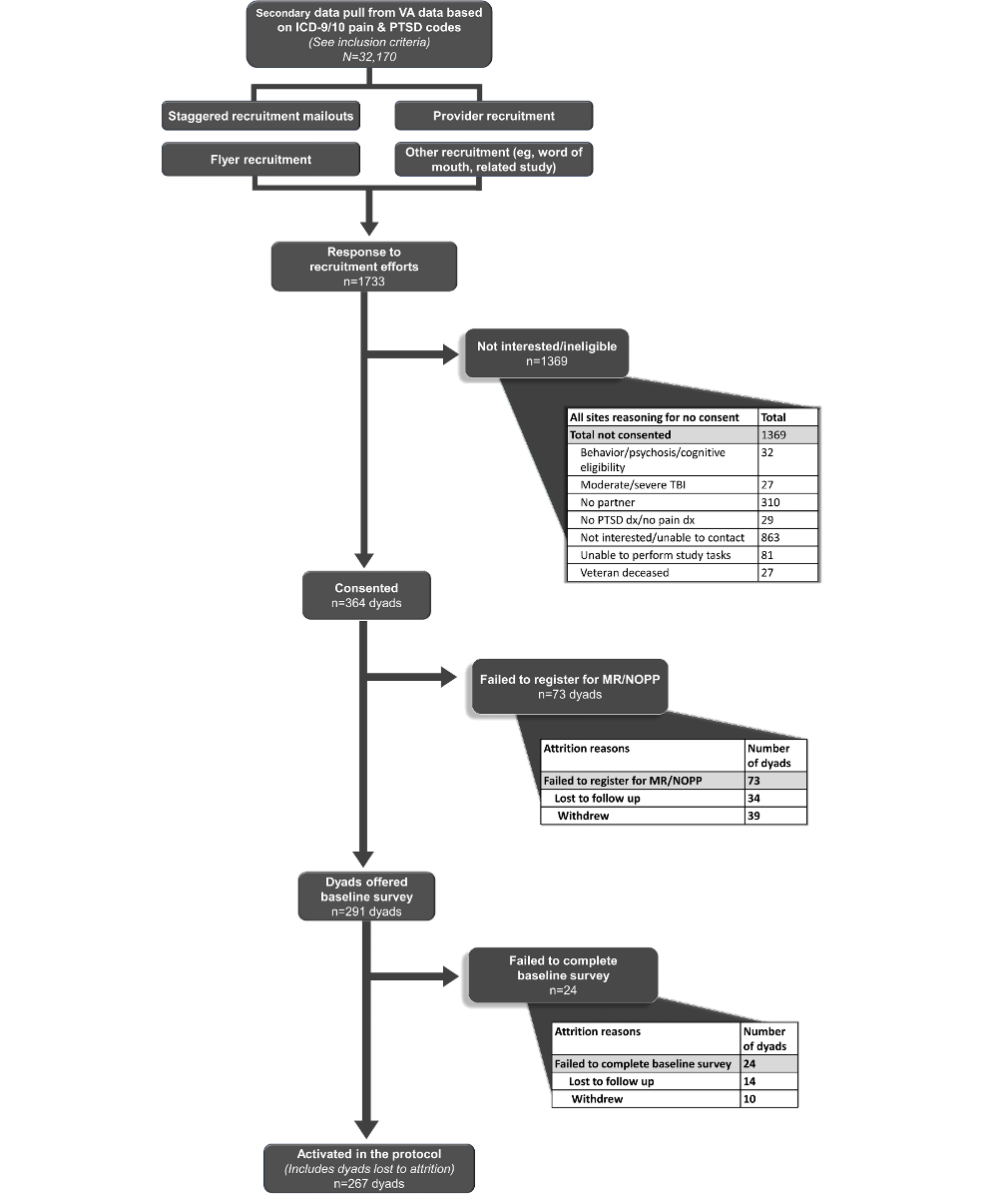
Mission Reconnect recruitment flow diagram. MR: Mission Reconnect; NOPP: notice of privacy practices; PTSD: posttraumatic stress disorder; TBI: traumatic brain injury.

Baseline dyad attrition occurred in 2 stages of the process to activate in the study protocol (see Figure 1). First, dyads could fail to complete MR onboarding or complete the NOPP. Second, dyads that registered for MR could fail to complete the baseline survey. Participants who “actively” withdrew from the study (verbal confirmation) were documented via off-boarding phone calls with participants. Participants who failed to respond to phone calls were categorized as “passively” withdrawn a week after the third outreach attempt. Participant dyads who failed to continuously engage and complete the MR program onboarding and the baseline survey were considered lost to follow-up (LTFU).

Qualitative data regarding reasons for attrition were gathered from a convenience sample of 10 veterans who passively withdrew or were LTFU. Actively withdrawn participants were not considered for qualitative interviews due to their request not to participate in this study. Participants were contacted via telephone and invited to engage in a brief attrition interview. With participants’ permission, interviews were audio-recorded and professionally transcribed. An interview guide (see Multimedia Appendix 4) was developed for attrition interviews.

### Measures

#### Screening Interview

Veterans were asked 7 self-report questions to determine eligibility criteria and randomization stratum including current diagnoses and recent or concurrent treatment (chronic pain and PTSD) verification. Three additional eligibility questions assessed self-reported moderate-to-severe traumatic brain injury (TBI), recent diagnosis or treatment for psychotic disorders, and current treatment for substance use disorders. Both veterans and partners were asked 3 questions about routine internet access, English-language fluency, and visual/hearing/cognitive issues that may interfere with the ability to consent. Finally, both veteran and partners completed 3 items regarding physical violence and aggression in the relationship. All screening items were binary (yes or no).

#### TBI Screen

Participants were screened for TBI using The Ohio State University TBI Identification Method [35]. This 8-item structured interview assesses self-report of head, neck, or high-impact injuries to elicit TBI across a participant’s lifetime. Follow-up probes are used on up to 3 of the most severe injuries. Participants who reported losing consciousness for 30 minutes to 24 hours (moderate TBI) or ≥24 hours (severe TBI) were excluded [36].

#### Participant Demographics

Demographic data for successfully enrolled participants were collected as part of the baseline Qualtrics survey.

#### Pain Intensity

A single-item 0-10 Numeric Rating Scale (NRS) from the Pain Outcomes Questionnaire-VA was used to assess pain intensity [37]. Veteran pain intensity was classified at baseline using established cutoffs for the 0-10 NRS: mild (≤3), moderate (4-6), and severe (≥7) [38].

#### Posttraumatic Stress Disorder

The 20-item PTSD Checklist for Diagnostic and Statistical Manual of Mental Disorders-Fifth Edition (PCL-5) [39] was used to classify “provisional” PTSD based on symptom severity. All items were measured from 0 (*Not at All*) to 4 (*Extremely*). To describe our sample at baseline, a lower bound cutoff score recommended by the VA National Center for PTSD was used to classify participants [40]. Participant scores of ≥31 on the PCL-5 suggest probable PTSD. Scores <31 on the PCL-5 indicate a subthreshold for meeting the criteria for PTSD.

#### Geographical Location

Veteran geographical location and rurality data were established using the primary physical address provided to receive study materials. Rurality was determined from the 2004 Rural Urban Commuting Area (RUCA) codes crosswalk file available from the University of Washington [41]. Veterans were classified as urban (RUCA code=1-3) or rural (RUCA code=4-10) using guidelines set by the VA Office of Rural Health [42].

### Data Analyses

#### Quantitative

Descriptive data are presented as number and percent for categorical data, and means and SDs for continuous data. Baseline comparisons between treatment arms were performed using chi-square tests, independent samples *t* tests, or Fisher exact tests. Descriptive analyses and group comparisons were conducted using SAS (version 9.4).

#### Qualitative

Interview transcript data were analyzed using descriptive content analysis methods to identify domains and taxonomies related to participants’ experiences with MR. Using Atlas.ti (version 9; ATLAS.ti Scientific Software Development GmbH), the initial 2 transcripts were coded by 3 qualitative researchers to devise a preliminary coding schema using an open-coding approach to identify reasons for participant attrition and recommendations to mitigate the occurrence of attrition. Once this preliminary schema was developed, the remaining transcripts were coded by 1 qualitative researcher.

## Results

### Initial Mailout Response

A prestudy data extraction from the VA CDW yielded 32,170 veterans with a diagnosis of both chronic pain and PTSD. Prospective veterans (n=1733, 5.4%) responded to recruitment mailers. Of these veterans, 1369 (4.3%) veterans were either ineligible or uninterested in study participation. Primary reasons for ineligibility included inability to contact or uninterested (n=863, 2.7%), no partner (n=310, 1%), unable to perform study tasks (n=81, 0.3%), psychotic disorder or cognitive difficulty (n=32, 0.1%), no pain or PTSD diagnosis (n=29, 0.09%), positive moderate-to-severe TBI screen (n=27, 0.08%), and veteran deceased (n=27, 0.08%). In total, 364 (1.1%) veteran-partner dyads consented for this study. A flow diagram of the veteran-dyad recruitment is presented in Figure 1.

### Geographic Recruitment of Veteran-Partner Dyads

**Figure 2:**
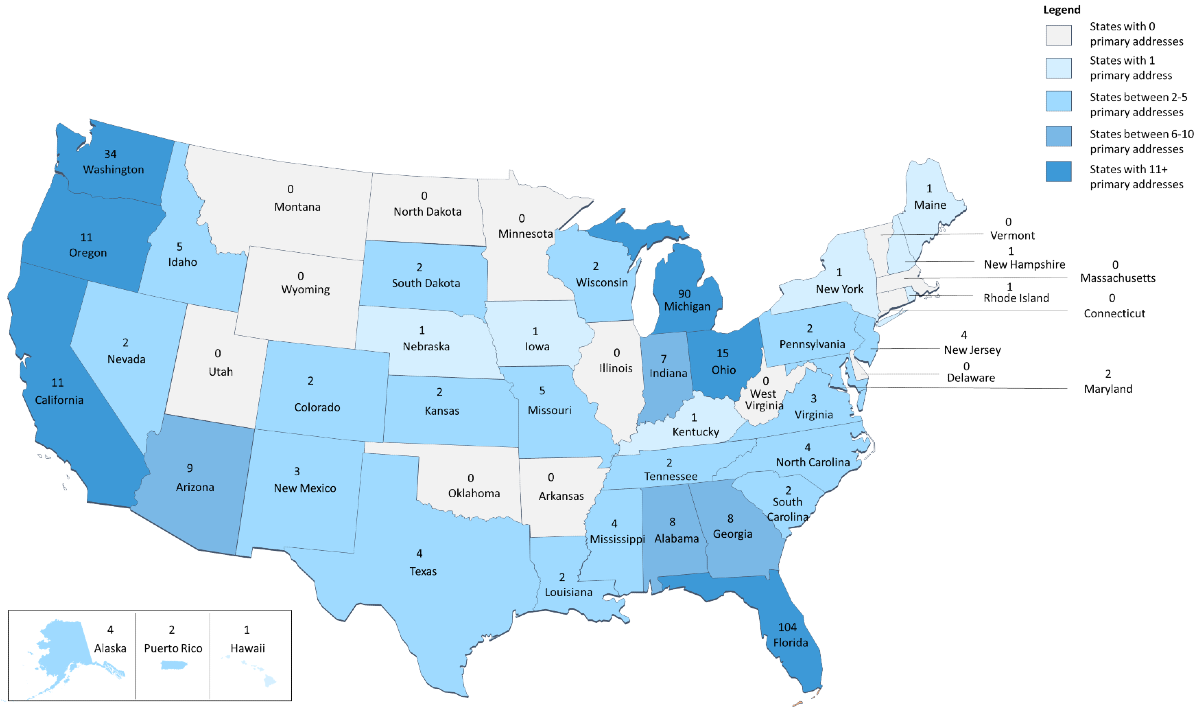
State and US territories with number of participants (n=363). Participants' location identified by primary mailing address.

The consented dyads (n=364) reflect the geographic diversity of our recruitment sample. Specifically, our recruitment efforts led to dyads being recruited from 37 US states and 1 territory (Puerto Rico). The majority of dyads were recruited from the states where VA study sites were located, including Florida (n=104, 28.6%), Michigan (n=90, 25%), and Washington (n=34, 9%). The additional 136 (37.4%) dyads were recruited from 35 US states and territories. Of the 364 recruited dyads, most heard about the study through the mailer (n=325, 89.3%). The remaining consenters learned via flyers (n=3, 0.8%), multiple methods (eg, flyer+mailer; n=3, 0.8%), and 33 (9%) reported “other” methods (eg, provider referral). Using RUCA codes and VA Office of Rural Health guidelines, we identified 298 (81.9%) dyads living in urban areas. The remaining 66 (18%) dyads identified as living in rural areas. A diagram of dyad recruitment by geographic location is presented in Figure 2.

### Attrition for Veteran-Partner Dyads

In total, 267 of 364 (73.4%) dyads activated the study protocol, and 97 (27%) dyads were lost to attrition. Regarding attrition, 73 (20%) dyads failed to register for MR or return NOPP. Self-reported reasons dyads were LTFU (n=34, 9%) included COVID-19 concerns (n=6, 2%), holiday season (n=4, 1%), personal time constraints (n=4, 1%), and 4 (1%) dyads listing separate other concerns (health issues, technical issues, frustration with study process, partner withdrawal). We were unable to determine the LTFU reason for 16 (4%) dyads. A total of 39 (11%) dyads withdrew from this study for the following reasons: time constraints with performing study activities (n=14, 4%), physical or emotional issues independent of the study (n=10, 3%), partner withdrawal (n=3, 0.8%), and 3 (0.8%) dyads that listed other reasons (COVID-19, privacy concerns, and conflict with another study). Nine (2%) dyads did not provide a reason for study withdrawal.

An additional 24 (7%) dyads completed MR registration but failed to complete the baseline survey. Fourteen (4%) of these dyads self-reported the following reason they were LTFU: possible technical issues (n=4, 1%), health issues (n=2, 0.5%), and partner withdrawal (n=2, 0.5%). We were unable to obtain a determining factor for the 6 (2%) remaining LTFU dyads. Finally, 10 (3%) dyads withdrew citing time constraints (n=4, 1%), physical or emotional health issues independent of study participation (n=3, 0.8%), partner withdrawal (n=2, 0.5%), or no reason was obtained (n=1, 0.3%).

### Demographic Characteristics

Two (0.5%) dyads completed both MR onboarding and the baseline survey but were excluded from this analysis. One dyad requested that we do not use their data after the study withdrawal. The second dyad left the study after the partner withdrew. The veteran and study team reached an agreement to discard these data because the veteran wanted to rejoin the study with a new partner, but this never came to fruition. These 2 dyads were not counted in baseline attrition rates because they completed the required onboarding and baseline survey procedures.

Baseline demographic characteristics from 265 veteran-partner dyads that completed MR onboarding and baseline survey procedures are reported. Veterans had an average age of 56.5 (SD 13.7) years. These participants were typically male (n=196, 74%), White (n=193, 72.8%), married (n=203, 76.6%), had an education level of associate’s or higher degree (n=175, 66%), and reported daily computer (n=162, 61.1%) and internet use (n=213, 80.4%). The self-reported length of relationship with their partner had a relatively similar distribution of <10 years (n=88, 33%), 10-29 years (n=96, 36%), and ≥30 years (n=79, 30%).

Partners had an average age of 52.6 (SD 14.1) years. These participants were typically female (n=217, 81.9%), White (n=202, 76.2%), married (n=208, 78.5%), had an associate’s or higher degree (n=150, 56.6%), and reported daily computer (n=174, 65.7%) and internet use (n=234, 88.3%). The self-reported length of the relationship varied slightly from that of the veteran and had a relatively similar distribution of <10 years (n=81, 31%), 10-29 years (n=103, 38.9%), and ≥30 years (n=79, 30%). No statistically significant demographic differences between study arms were observed for veterans or partners. Demographic information for veterans and partners across study arms is presented in Table 1.

**Table 1:** Baseline demographic characteristics of participant dyads in the Mission Reconnect (n=133 dyads) and waitlist control (n=132 dyads) arms.

Characteristic			Veterans^a^								Partners^a^						
			Mission Reconnect		Waitlist control		*P*^b^ value			Mission Reconnect			Waitlist control			*P*^b^ value	
Age (years), mean (SD)			55.8 (14.1)		57.3 (13.4)		.38			52.2 (13.7)			52.9 (14.6)			.68	
**Gender,** **n** **(%)**								.83									.11
	Female	35 (26.3)		33 (25)					103 (77.4)			114 (86.4)					
	Male	97 (72.9)		99 (75)					29 (21.8)			17 (12.9)					
	Other	1 (0.8)		0 (0)					1 (0.8)			1 (0.8)					
**Race, n (%)**								.93									.99
	White	97 (72.9)		96 (72.7)					101 (75.9)			101 (76.5)					
	African American or Black	18 (13.5)		20 (15.2)					19 (14.3)			21 (15.9)					
	Asian	1 (0.8)		1 (0.8)					5 (3.8)			4 (3)					
	American Indian or Alaska Native	3 (2.3)		1 (0.8)					1 (0.8)			0 (0)					
	Multiracial	9 (6.8)		8 (6.1)					3 (2.3)			2 (1.5)					
	Native Hawaiian or Pacific Islander	0 (0)		0 (0)					1 (0.8)			1 (0.8)					
	Other	4 (3)		6 (4.6)					2 (1.5)			1 (0.8)					
	Missing or decline to respond	1 (0.8)		0 (0)					1 (0.8)			2 (1.5)					
**Hispanic ethnicity,** **n** **(%)**								.43									.94
	Hispanic or Latino	13 (9.8)		9 (6.8)					11 (8.3)			9 (6.8)					
	Non-Hispanic or Latino	115 (86.5)		114 (86.4)					119 (89.5)			120 (90.9)					
	Missing or decline to respond	5 (3.8)		9 (6.8)					3 (2.3)			3 (2.3)					
**Marital status,** **n** **(%)**								.27									.75
	Married or partnered	107 (80.5)		96 (72.7)					108 (81.2)			100 (75.8)					
	Divorced, separated, or widowed	22 (16.5)		32 (24.2)					12 (9)			15 (11.4)					
	Single/never married	4 (3)		3 (2.3)					12 (9)			15 (11.4)					
	Missing or decline to respond	0 (0)		1 (0.8)					1 (0.8)			2 (1.5)					
**Education,** **n** **(%)**								.76									.39
	Less than high school	0 (0)		0 (0)					0 (0.0)			1 (0.8)					
	High school	11 (8.3)		6 (4.6)					18 (13.5)			23 (17.4)					
	Some college or vocational school	34 (25.6)		39 (29.6)					42 (31.6)			30 (22.7)					
	Associate degree	31 (23.3)		29 (22)					20 (15)			27 (20.5)					
	Bachelor degree	32 (24.1)		33 (25)					32 (24.1)			27 (20.5)					
	Graduate degree	25 (18.8)		25 (18.9)					21 (15.8)			23 (17.4)					
	Missing or decline to respond	0 (0)		0 (0)					0 (0)			1 (0.8)					
Daily computer use, n (%)			85 (63.9)		77 (58.3)		.39			91 (68.4)			83 (62.9)			.39	
Daily internet use, n (%)			107 (80.5)		106 (80.3)		.92			116 (87.2)			118 (89.4)			.58	
**Years in relationship with a partner (years), n (%)**								.44									.47
	<10	47 (35.3)		41 (31.1)					43 (32.3)			38 (28.8)					
	10-29	46 (34.6)		50 (37.9)					50 (37.6)			53 (40.2)					
	≥30	38 (28.6)		41 (31.1)					38 (28.6)			41 (31.1)					
	Missing or decline to respond	2 (1.5)		0 (0)					2 (1.5)			0 (0)					

^a^Percentiles may not equal 100% exactly secondary to rounding error.

^b^*P* values obtained from *t* test, chi-square, or Fisher exact tests.

Using established scale cutoffs, veteran participants were more likely to report moderate (n=119, 44.9%) or severe (n=125, 47.2%) pain intensity at baseline. Most veterans were categorized as having probable PTSD (n=200, 75.5%). Veteran chronic pain and PTSD classifications are presented in Table 2.

**Table 2:** Mission Reconnect baseline classifications of pain intensity and posttraumatic stress disorder in the veteran sample (n=265).

		PTSD cutoff^a,b^		Total
		Subthreshold (<31)	Probable (≥31)	
**Pain intensity cutoff^c^, n (%)**				
	Mild (<3)	14 (5.2)	7 (2.6)	21 (7.9)
	Moderate (4-6)	32 (12.1)	87 (32.8)	119 (44.9)
	Severe (≥7)	19 (7.2)	106 (40)	125 (47.2)
	Total	65 (24.5)	200 (75.5)	265 (100)

^a^The PTSD Checklist for Diagnostic and Statistical Manual of Mental Disorders-Fifth Edition (PCL-5) was used with a validated cutoff for identifying probable PTSD among veterans. Scores <31 on the PCL-5 indicate subthreshold or no PTSD. PCL-5 scores ≥31 suggest probable benefit from PTSD treatment.

^b^PTSD: posttraumatic stress disorder.

^c^Pain intensity assessed using the 0-10 Numeric Rating Scale and categorized using a valid cutoff.

### Qualitative Attrition Interviews

Attrition (vs LTFU) was most likely to occur for participants who did not successfully complete registration for MR. Because the qualitative attrition interviewees were randomly selected, the subsample (n=10) is theoretically similar to the overall attrition group. Although varied reasons were identified for participant attrition, interviewees predominately identified the construct of “personal circumstances.” Veterans and their partners indicated circumstances in their lives such as competing personal health concerns or death in the family as factors that pulled their attention from the MR onboarding process. This construct is exemplified by 1 participant stating, “I had some health issues and stuff come up on our plates that we weren’t able to fulfill our side of the agreement or the program.”

Interviewees also indicated a lack of understanding of MR as a program. Interviewees reported confusing the weekly research surveys as the intervention or not understanding the program’s intent. One veteran stated, “I don’t feel no (sic) connection with all these questions and the title of the program.” Excess paperwork was also identified as a factor contributing to participant attrition. Interviewees perceived the mailed informational and registration packet as overwhelming, “too tedious,” or they did not prioritize completing the forms. In addition to exploring the reason for attrition, interviews also inquired about potential means to improve the study experience. The most reported recommendation was to increase human interaction. Some interviewees stated preferences for person-to-person interaction to provide program assistance in times of need and maintain study engagement. One veteran stated “I need somebody to hold my hand” when it comes to MR onboarding. Additionally, interviewees recommended digital forms to minimize the use of paper and decrease potential feelings of burden associated with completing the forms. The latter recommendation also addresses participant concerns with the forms being overwhelming.

Participants often cited similar reasons for withdrawing from the study onboarding process. Passive and active withdrawals cited partners that withdrew after consenting, difficulties experienced with the mobile and web-based platforms for data collection and the intervention, and adverse health issues that were unrelated to study activities.

### Challenges and Recommendations for Study Recruitment

Study recruitment and onboarding are foundational to the success of a study. Based on experiences with this study, the study team developed a list of challenges and recommendations (Table 3).

**Table 3:** Challenges and recommendations to enhance study recruitment and onboarding.

Challenges	Recommendations
Need proactive approach to working with participants with pain and PTSD^a^	Document and track barriers and solutions for institutional review board and funding agency Pilot protocol early in the project to make modifications to onboarding, data collection, and processes to meet participants’ needs
SI^b^ more common than general population	Increase ceiling on SI reporting—change SI trigger from thoughts to plan and intent Consider a dedicated clinical psychologist to screen for SI and respond to reported participant mental health needs
Withdraw from studies at higher rates than the general population	Set realistic expectations Simplify and ensure a user-friendly onboarding process Proactively identify health factors and disqualifiers in advance; refer to published studies for guidance
Increased risk of frustration with onboarding and data collection processes	Simplify the onboarding process and provide personal support Increase automation where possible and reduce the use of usernames and passwords Color envelopes or add study-specific stamps to differentiate study-mailed materials from other VA^c^-mailed materials
Potential perceived data collection burden	Be cognizant of emotional, mental, and physical health burdens Revisiting trauma as a risk factor Use validated measures but also avoid multiple measures to reduce redundancy to minimize response burden Digitize onboarding paperwork for payment processing and inclusion of caregiver dyads as participants
Lack of engagement in project processes	Simplify processes Provide reminders Provide a project navigator

^a^PTSD: posttraumatic stress disorder.

^b^SI: suicidal ideation.

^c^VA: Department of Veterans Affairs.

## Discussion

### Principal Results

This study describes the recruitment, onboarding, and attrition in a remotely delivered RCT examining MR. Although recruitment occurred at 3 sites in separate states (Florida, Michigan, and Washington), veteran and partner dyads who responded to recruitment efforts represented 37 US states and 1 territory (Puerto Rico), resulting in a more geographically diverse sample than anticipated. In addition, ICD-10 codes for chronic pain and PTSD were used as sampling criteria for targeted recruitment efforts, which resulted in a sample that reported moderate to severe pain (n=244, >92%) and probable PTSD (n=200, >75%) on the NRS and PCL-5, respectively. Key findings regarding study attrition were that our roughly 27% (97/364) attrition rate exceeded the 20% (46/228) anticipated rate but was still within an acceptable range for chronic pain and PTSD trials [18-20,23,24,43]. Attrition interviews revealed that participants did not complete MR registration for several reasons including misunderstanding the intent of MR, mistaking the survey completion for the intervention, personal circumstances that interfered with participation (eg, health concerns), and study mailer packets being too tedious and confusing. Common reasons for attrition following MR registration included partner withdrawal, health concerns unrelated to MR, and technological challenges with using MR and Qualtrics. Finally, recommendations for reducing attrition included switching to digital forms to reduce burden and increasing human interaction through the onboarding and baseline data collection processes.

As researchers support efforts to examine nonpharmacological interventions to help individuals manage pain and PTSD, effective delivery of remote CIH interventions is shaped by symptom burden, accessibility of web-based programs, and population characteristics. A rigorous assessment of these factors is imperative to tailor research and programs made accessible for this population. This paper describes attrition during the recruitment, onboarding, and baseline data collection stages of an RCT assessing the efficacy of the MR intervention for veterans with comorbid chronic pain and PTSD and their partners. Examining baseline attrition and influencing factors provides useful enrollment recommendations and lessons learned to inform successful recruitment and retainment in future research and programmatic recruitment efforts specific to remotely delivered interventions designed for this clinically diverse patient population.

First, and notably, CDW sampling for contact and eligibility criteria, based on inclusion criteria, to identify eligible participants, resulted in a 5.4% (n=1733) response rate, with a recruitment rate of 1.1% (n=364). These values provide a reasonable expectation of sampling for protocols and interventions of a similar nature [14]. Notably, over 90% (n=244) of the veteran sample reported moderate-to-severe chronic pain intensity on an NRS, and over 75% (n=200) reported probable active PTSD, using the PCL-5 at baseline. These rates reflect the anticipated symptomology for veterans seeking CIH interventions to manage comorbid chronic pain and PTSD.

The geographic diversity of remote recruitment of veteran-partner dyads resulted in a national sample 34 US states and 1 territory, which highlights the opportunity for increasing access when conducting remote interventions. Mailings were clearly optimal for geographically diverse recruitment and using RUCA codes created an opportunity to target rural audiences. Overall, baseline demographic characteristics for dyads were reflective of the larger veteran and caregiver population. One notable factor to consider when recruiting for mobile interventions is that most of this population had higher educational levels and daily computer and internet use. This accounts for a self-selection bias based on educational, and computer and internet literacy.

Overall, the study attrition rate was about 27% (n=97); we retained over 73% (n=267) of dyads through activation which is standard, based on rates reported in the literature for chronic pain, PTSD, and web-based dyadic interventions [18-20,23,24,43]. Two primary processes that created pain points were onboarding and completing baseline data collection. The most common reason for failure to successfully onboard was because they did not complete the intervention registration; however, attrition interviews did not elicit any predominant reason for failure to register, other than feeling overwhelmed by the paperwork and logistical processing. Although the paperwork and data collection were electronic, recommendations were made to make paperwork for payment processing and inclusion of caregiver dyads as participants electronic as well.

The second most common reason for attrition was active withdrawal—which was cited as time constraints to perform study activities—often related to other competing personal priorities, such as health and family priorities. Identified recruitment and attrition issues were identified early in the project; when possible, iterative approaches were used to adapt the protocol and processes to meet participant needs.

Attrition interviews provided some insight into attrition as well; for example, attrition interview participants reported a lack of understanding of the purpose of the MR program. It is important to note that though this project was innovative in its use of remote technology and strategies and was only able to continue during COVID-19 due to its use of remote technology, the most reported recommendation was to increase human interaction. As systems turn to remote access in a post COVID-19 climate, this trial was an opportunity to learn the need to balance remote accessibility with human contact, as technology and remote access to care, though valuable and sometimes necessary, leave the risk of leaving patients feeling disconnected. Lack of program usefulness and awareness of potential benefits, lack of the technical or necessary equipment to participate, or not having a partner to participate in the intervention are programmatic issues that require further in-depth analysis for improving programmatic implementation.

While higher than our initial expectations, our final attrition rate is consistent with studies that recruit veterans with chronic pain and PTSD. While previous studies have identified specific barriers for veterans with pain and PTSD seeking treatment for symptom management, these barriers often were associated with in-person treatment. The similar attrition rates reveal either parallel or overlapping factors that make it difficult for veterans to participate in remote interventions for symptom management. Although participant attrition for the MR trial was commiserated with published rates [18-20,23,24,43], there are still lessons to be learned and opportunities to mitigate cited reasons for attrition in future efforts. First, it is recommended to separate the study process during the onboarding phase from the actual intervention and the data collection as these are specific areas of focus, which will benefit from proactive approaches throughout the study implementation process. Notably, user experiences with electronic platforms, and adverse health issues, are generally unrelated to study activities. This is an important distinction as it informs the readiness of this population to engage in (1) remotely delivered interventions, (2) technology platforms, (3) research protocols designed to address pain and PTSD, and (4) partnered interventions. As researchers and program developers continue to develop, test, and implement nonpharmacological CIH to manage pain and PTSD, the complexities and elements of remotely delivered partnered web-based interventions cannot be underestimated as determinants that can impact participant engagement.

### Limitations

Multiple study limitations should be noted. First, baseline data collection was the final step of the MR onboarding process and we were unable to collect demographic information from participant dyads that failed to complete MR onboarding or the baseline survey. This restricted our capability to make meaningful comparisons between dyads that completed the MR intervention baseline and the attrition subsample. Future studies should consider the opportunity to collect demographic data for the exploration of diversity factors and social determinants of health as potential factors impacting attrition. Second, the MR intervention was dyadic and only available for veterans who have a partner. The dyadic requirement may have contributed to the attrition rate and reduced veteran response rate to initial recruitment efforts. Future research opportunities include examining nonpartnered options for MR and similar interventions. Third, self-report studies may be subject to the Hawthorne effect and participants providing socially desirable responses due to knowledge of being observed. Fourth, targeted recruitment methodologies primarily relied on ICD-10 codes for chronic pain and PTSD diagnoses. However, there is notable between-provider variability in both chronic pain [44] and PTSD [45] diagnoses based on ICD-10 classifications. Future studies can benefit from additional precision of their chronic pain and PTSD sampling criteria via chart review of participants’ chronic pain and PTSD presentation against additional standardized diagnostic criteria (eg, Diagnostic and Statistical Manual for Mental Disorders). Fifth, and perhaps most important, this remote study protocol was conducted during the COVID-19 pandemic. This resulted in unique circumstances that resulted in losing participants during the onboarding process as revealed during attrition interviews. Future interventions conducted postpandemic may experience lower attrition rates.

### Conclusions

The remote recruitment, onboarding, and data collection processes made this protocol potentially challenging for medically complex populations managing chronic pain and PTSD, warranting special considerations for future trials. Increasing access to nonpharmacological interventions can play a critical role in helping individuals manage pain and PTSD. Disseminating recruitment and attrition challenges, solutions, and lessons learned are critical to informing best practices when addressing the unique needs of medically complex patient populations when delivering remote self-directed CIH interventions. Successful enrollment of veterans with chronic pain and PTSD, and their partners, to remote CIH treatments requires future examination of demographic and symptom-associated access barriers. Strategies for accommodating barriers are essential for improving the effectiveness of CIH programs. Characteristics of successfully enrolled participants inform target populations who can be best served by remote CIH. Disseminating lessons learned and improving access to remotely delivered CIH programs is paramount in the COVID-19 climate.

## Appendix

Multimedia Appendix 1 CONSORT-EHEALTH (Consolidated Standards of Reporting Trials of Electronic and Mobile Health Applications and Online Telehealth) checklist.

Multimedia Appendix 2 Promotional flyer for Mission Reconnect recruitment.

Multimedia Appendix 3 Inclusion and exclusion criteria for veteran-partner dyad responses to recruitment efforts.

Multimedia Appendix 4 Mission Reconnect interview guide.

## Abbreviations

CDW: Corporate Data Warehouse
CIH: complementary and integrative health
CONSORT-EHEALTH: Consolidated Standards of Reporting Trials of Electronic and Mobile Health Applications and Online Telehealth
ICD-10: International Classification of Diseases-10th Edition
LTFU: lost to follow-up
MR: Mission Reconnect
NOPP: notice of privacy practice
NRS: National Rating Scale
PCL-5: PTSD Checklist for Diagnostic and Statistical Manual of Mental Disorders-Fifth Edition
PRO: patient-reported outcome
PTSD: posttraumatic stress disorder
RCT: randomized controlled trial
RUCA: Rural Urban Commuting Area
TBI: traumatic brain injury
VA: Department of Veterans Affairs

## Data Availability

Deidentified anonymized data sets depicted in this paper are available from the corresponding author upon reasonable request.
