# Supplementary material for: Mobile and Web-Based Partnered Intervention to Improve Remote Access to Pain and Posttraumatic Stress Disorder Symptom Management: Recruitment and Attrition in a Randomized Controlled Trial
Source: J Med Internet Res. 2023 Oct 3;25:e49678. doi: 10.2196/49678 (PMC10582813; doi:10.2196/49678)
Supplement: Multimedia Appendix 2 [file jmir_v25i1e49678_app2.pptx]

## Slide 1
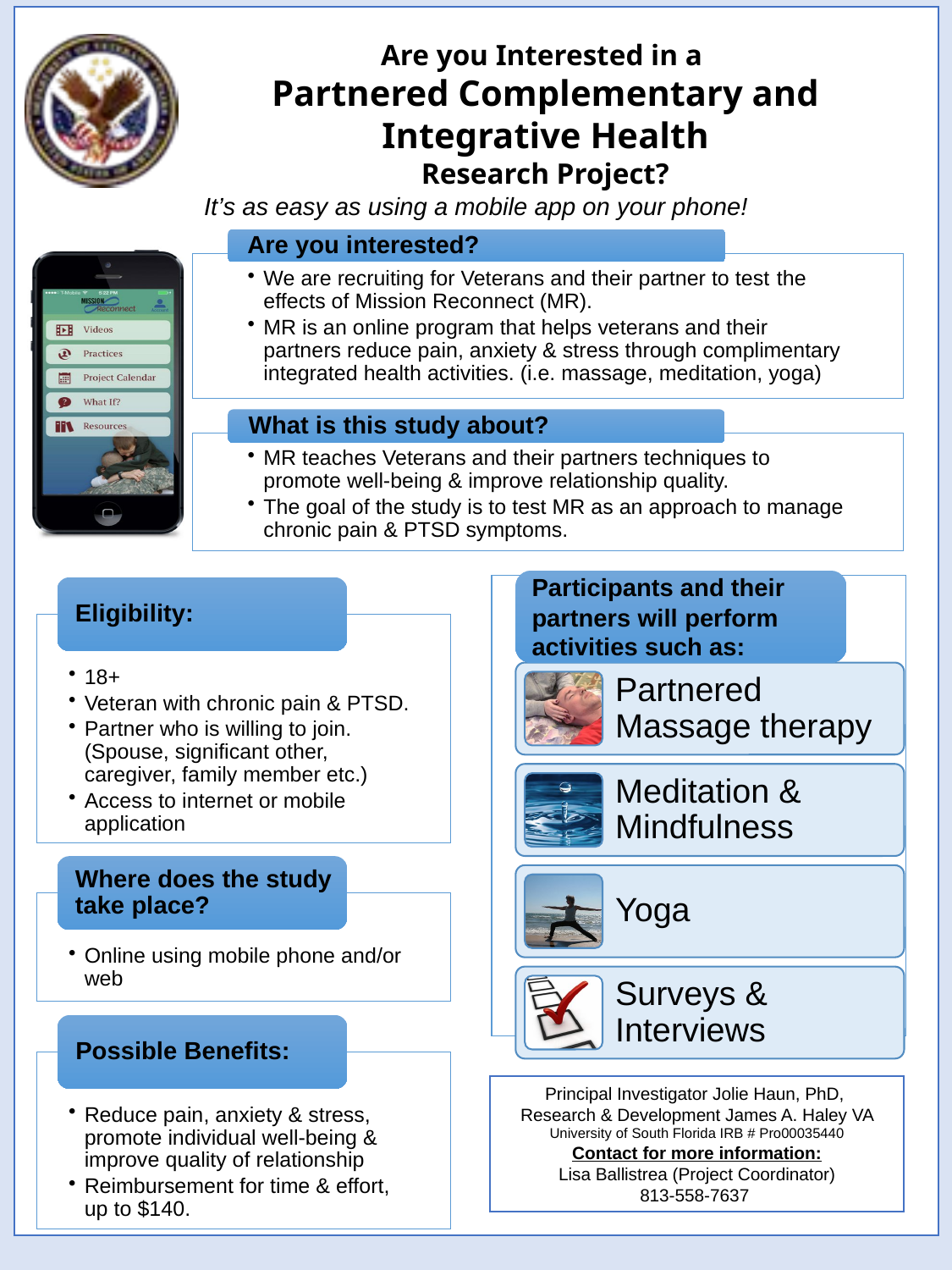

Are you Interested in a
Partnered Complementary and Integrative Health
Research Project?
It’s as easy as using a mobile app on your phone!
Are you interested?
We are recruiting for Veterans and their partner to test the effects of Mission Reconnect (MR).
MR is an online program that helps veterans and their partners reduce pain, anxiety & stress through complimentary integrated health activities. (i.e. massage, meditation, yoga)
What is this study about?
MR teaches Veterans and their partners techniques to promote well-being & improve relationship quality.
The goal of the study is to test MR as an approach to manage chronic pain & PTSD symptoms.
Participants and their partners will perform activities such as:
Principal Investigator Jolie Haun, PhD,
Research & Development James A. Haley VA University of South Florida IRB # Pro00035440
Contact for more information:
Lisa Ballistrea (Project Coordinator)
813-558-7637
