# Supplementary material for: Mobile and Web-Based Partnered Intervention to Improve Remote Access to Pain and Posttraumatic Stress Disorder Symptom Management: Recruitment and Attrition in a Randomized Controlled Trial
Source: J Med Internet Res. 2023 Oct 3;25:e49678. doi: 10.2196/49678 (PMC10582813; doi:10.2196/49678)
Supplement: Multimedia Appendix 3 [file jmir_v25i1e49678_app3.docx]

Appendix 3: Eligibility and Ineligibility Criteria for Study Participation

| **Eligibility Criteria** | |  |
| --- | --- | --- |
| **Criteria** | **Measure** | **Applicable Dyad Member** |
| Co-occurrence of Chronic Pain and Post-traumatic Stress Disorder (PTSD) | - Collected though extraction of medical data from the VA’s Central Data Warehouse (CDW). Participants identified by presence of 2 targeted musculoskeletal pain ICD-10 codes, separated by 30 days, and a PTSD ICD-10 code. Extraction occurred for time period 6 months prior to initiation of recruitment activities. - Verified by participant self-report of diagnosis. | Veteran |
| Age 18+ | - Verified through medical records and participant self-report. | Veteran/Partner |
| English-speaking Veterans | - Verified through participant screening process and participant self-report. | Veteran/Partner |
| A partner to perform study activities | - Identified during the screening process. Partner and Veteran consented contemporaneously during onboarding. | Veteran/Partner |
| **Ineligibility Criteria** | |  |
| **Ineligibility Criteria** | **Measure** | **Applicable Dyad Member** |
| Moderate to Severe Traumatic Brain Injury (TBI) | - Excluded based on targeted ICD-10 codes. - Suspected cases screened with the Ohio TBI Screen. | Veteran |
| Diagnosis or Treatment for psychosis in the 6 months prior to initiating study activities | - Excluded based on targeted ICD-10 codes. - Verified though chart screens and participant self-report. | Veteran |
| Currently enrolled in a formal substance-abuse treatment program | - Excluded based on targeted ICD-10 codes. - Verified though chart screens and participant self-report. | Veteran |
| Visual, hearing, or cognitive impairment that prevents participation and/or ability to consent | - Excluded based on targeted ICD-10 codes. - Verified though chart screens and participant self-report. | Veteran/Partner |
| Lack of Access to Internet and/or Virtual elements of Study | - Verified through screening process, and access. verified by registration for online tools during the on-boarding process. | Veteran/Partner |
| Suspected Domestic Violence and/or Aggression | - Both partners are individually screened using the Partner/Family Member Abuse Screen used in VA Family Services. | Veteran/Partner |
