# Supplementary material for: Mobile and Web-Based Partnered Intervention to Improve Remote Access to Pain and Posttraumatic Stress Disorder Symptom Management: Recruitment and Attrition in a Randomized Controlled Trial
Source: J Med Internet Res. 2023 Oct 3;25:e49678. doi: 10.2196/49678 (PMC10582813; doi:10.2196/49678)
Supplement: Multimedia Appendix 4 [file jmir_v25i1e49678_app4.docx]

# Appendix 4

**Mission Reconnect: Phone Interview Script for Veterans and Partners**

Hello, may I speak to (Name of Participant)? (If Participant not in, record a time for calling back.)

Hi, my name is (interviewer name). I’m calling regarding the study “Mission Reconnect: Delivering a Mobile and Web Based Self Directed Complementary and Integrative Health Program to Veterans and Their Partners to Manage Pain and PTSD”. If you’ll remember, this study relates to the Mission Reconnect project you participated in. Is this a good time to talk?

YES, continue.

NO, schedule a time to call back.

I want to remind you why we are calling you. When we last spoke, I informed you about the study, received your initial consent, and you agreed to participate in a telephone interview if selected. I would like to inform you that you were selected for the telephone interview. The interview will take approximately XX minutes and will be conducted over the phone at the time convenient for you. We will ask questions about your experience participating in the Mission Reconnect program. I will also ask you a few questions about the program materials; your impressions; what you learned from the program; whether you have been able to apply what you learned; and any recommendations you may have for improving the program. Do you agree to participate in a telephone interview?

YES, continue.

NO, thank you for your participation in the study.

We can conduct the telephone interview now, if this is a good time. YES, continue.

NO, schedule a time to conduct the interview.

Before we begin the interview, I would like to turn on my audio recorder to get a recording of your consent to audio record. May I turn on the audio recorder?

YES, turn on audio recorder.

NO, thank you for your participation in the study.

The recording will be used to fill in my notes during the interview and help with accuracy of information. Do I have your permission to audio record the interview?

I would like to begin by thanking you for agreeing to do this interview with me. The purpose of the interview is to find out about your experiences as a participant in the Mission Reconnect program. We’re hoping to gain insight into your thoughts about the program. Findings from this interview and others like it will inform us about ways to better serve veterans and their partners. We value your opinion. Your input is essential to the successful implementation of Mission Reconnect.

Before proceeding I want to remind you that your participation is voluntary, meaning that you can end your participation in the interview at any time or refuse to answer any questions asked of you. If you decide not to complete this interview, your decision will not result in adverse consequence. Any information that you provide will be kept confidential. None of your comments will be reported publicly in a way that would identify you as the informant. Nor will your answers affect your health care services at the VA.

Only members of the research team will have access to the information discussed in the interview. During the interview, we will use our first names only to protect your privacy. Please be assured that there is no right or wrong answer. We want to know about your experiences.

Do you have any questions?

(If YES, questions are addressed as applicable) If NO, Great! Let’s begin:

**Interview Guide A** (for participants who have been actively engaged in Mission Reconnect)

1. Let’s just start off by telling me about your experience participating in the Mission Reconnect Program?

**Probe-** In light of recent events, how is Mission Reconnect influencing your health and well-being?

1. How are you using the tools/information you learned from the Mission Reconnect program in your daily life?

| **Component** | **Activities** | **Probes/Notes** |
| --- | --- | --- |
| **Program Overview** | | - What was your experience with the program overview content? - Is there anything we can do to make this content more useful to you? |
| **Connecting with Yourself** | Morning Gratitude | - How often do you use them? - What was your experience using this activity? - (If no use) Is there something we could do to make this activity more useful to you? |
|  | Mirror Greeting |  |
|  | Loosening and Relaxing |  |
|  | Waking Up the Body |  |
|  | Reset and Refresh |  |
| **Connecting with Quiet** | Centering |  |
|  | Movement Into Stillness |  |
|  | Deep Relaxation |  |
| **Connecting with Your Partner** | Seeing Each Other |  |
|  | Giving Massage |  |
|  | Receiving Massage |  |
| **Support materials** | | - What was your experience with the support materials? - Is there anything we can do to make the support materials more useful to you? |

# Probes:- Are there any barriers/things that prevent you from using what you

# learned from the Mission Reconnect Program?

**Probes**: - Time? Confidence using the program?

- Partner commitment?

1. **[Veteran only]** Now that we reviewed your use of the program, can you talk to me about how using the Mission Reconnect program affected your pain experience? How has using the Mission Reconnect program affected your PTSD experience? (prompt: coping with pain, managed pain)

**Probes**: How would you compare your experience with pain before and after using the Mission Reconnect program? How would you compare your experience with PTSD before and after using the Mission Reconnect program?

1. What would help you better use the information and skills you learned from the Mission Reconnect program?

**Probes**: Different program structure?

Different materials?

Different information?

4a. **[Veteran only]** What would help you better use the information and skills you learned from the Mission Reconnect program to manage your pain and PTSD?

**Probes**: Different program structure?

Different materials?

Different information?

1. How would you like to see Mission Reconnect made available to users like you in the VA system?

**Probes**: Would all Veterans benefit from this program, if yes, how so?

How could this program best be made available to veterans with pain and PTSD?

1. Do you have any other recommendations of how we can improve the Mission Reconnect program?

“Thank you for discussing your experiences about the Mission Reconnect program. You have provided some great feedback. We really appreciate your time. Do you have any other comments before we conclude our interview today?”

(If YES, questions are addressed as applicable)

“Ok great, again thank you for your time and valuable feedback. Have a nice day, goodbye.”

**Interview Guide B** (for participants who may not have been engaged in Mission Reconnect)

1. Initially, what made you choose to participate in the Mission Reconnect project?
2. Can you tell me about your experience from the time you consented to participate to the point at which you engaged [or did not engage] in the Mission Reconnect project?
3. [If DID NOT use MR] What are some reasons you decided to not participate in Mission Reconnect activities?

**Probe:** What would have helped you use Mission Reconnect?

1. Do you have recommendations of how we can improve the Mission Reconnect project?

“Thank you for discussing your experiences about the Mission Reconnect project. You have provided some great feedback. We really appreciate your time. Do you have any other comments before we conclude our interview today?”

(If YES, questions are addressed as applicable)

“Ok great, again thank you for your time and valuable feedback. Have a nice day, goodbye.”
